# Serum parameters of inflammatory markers as prognostic biomarkers with maternal-neonatal outcome in patients with GDM

**DOI:** 10.3389/fmed.2024.1406492

**Published:** 2024-06-24

**Authors:** Xiaoyan Xiu, Yingying Lin, Zhiwei Chen, Lin Lin, Yizheng Zu, Jianying Yan

**Affiliations:** ^1^College of Clinical Medicine for Obstetrics & Gynecology and Pediatrics, Fujian Medical University, Fuzhou, China; ^2^Department of Obstetrics, Fujian Maternity and Child Health Hospital, Fuzhou, China; ^3^Department of Healthcare, Fujian Maternity and Child Health Hospital College of Clinical Medicine for Obstetrics & Gynecology and Pediatrics, Fujian Medical University, Fuzhou, China

**Keywords:** gestational diabetes mellitus, maternal-neonatal, outcome, inflammatory markers, serum parameters

## Abstract

**Objective:**

Gestational diabetes mellitus (GDM) is one of the most common complications of pregnancy, which is increasing annually. GDM can cause serious harm to both the mother and the offspring. However, the clinical indicators that predict pregnancy outcomes with GDM remain limited.

**Methods:**

This study included 3,229 pregnancies. Inflammatory markers were defective in the mother’s peripheral blood. Also, the Chi-square test, logistic regression analyses and Spearman rank correlation coefficient were performed to evaluate inflammatory markers with pregnancy outcomes. The association between inflammatory markers and pregnancy outcomes was analyzed. The optimal cut-off values of inflammatory markers were calculated.

**Results:**

Finally, 3,229 women were included. 1852 (57.36%) participants suffered good pregnancy outcomes. This study revealed that the maternal age, the baseline BMI (kg/m^2^), the times of parity, and the level of lymphocyte, SII and SIRI significantly increased in poor pregnancy outcomes groups. Additionally, inflammatory markers, such as white blood cells (WBC), neutrophils, monocytes, platelet counts, lymphocytes, systemic immune-inflammation index (SII) and systemic inflammation response index (SIRI) were related to pregnancy outcomes. Furthermore, the results revealed that the SII level had the highest odd rates (OR) [OR = 6.957; 95% CI (5.715–8.468)], followed by SIRI level [OR = 2.948; 95% CI (2.382–3.649)], the WBC counts [OR = 1.930; 95% CI (0.901–2.960)], the lymphocyte counts [OR = 1.668; 95% CI (1.412–1.970)], and baseline BMI [OR = 1.050; 95% (1.021–1.080)].

**Conclusion:**

This study presented that the baseline SII and SIRI levels can be valuable biochemical markers to predict the pregnancy outcome with GDM with non-invasive procedures. They can help identify high-risk pregnant women with GDM early, provide a personalized intervention in time, and enhance perinatal surveillance.

## Introduction

Gestational diabetes mellitus (GDM) is an abnormal glucose tolerance during pregnancy firstly, which is one of the most common complications of pregnancy (1). The global incidence of GDM ranges from 2.4 to 22.3%, and the average incidence of GDM in China is 14.8%, ranking among the top in the world (2–4). In recent years, the increase of elderly pregnant women because of the opening of the “two-child policy” and the improvement of China’s economic level, the number of obesity and diabetes people is gradually increasing. Related Meta-analysis shows that the prevalence of GDM among overweight and obese pregnant women can be as high as 30.3 and 26.7% among elderly pregnant women (3). The increasing incidence rate yearly has brought tremendous pressure and challenges to our health system.

GDM can cause serious harm to both the mother and the offspring at all stages. The risk of congenital malformation is 1.86 times that of non-GDM pregnancy, which may lead to abnormal fetal cardiovascular system, digestive tract, urinary system and central nervous system (5). What is more, GDM can cause myocardial structure and function of pregnant women assessed by speckle tracking echocardiography at an early and/or subclinical stage (6, 7). In the middle and third trimesters of pregnancy, shoulder dystocia and cesarean delivery risk were 2.74 times and 1.46 times more than non-GDM women, respectively (8, 9). The risk of macrosomia and the neonatal-perinatal mortality rate was 3.43 times and two times that of non-GDM pregnancies, respectively (8, 9). The offspring of GDM mothers had a significantly increased risk of obesity or metabolic syndrome, with an eight times higher risk of prediabetes or diabetes in adulthood than the normal population (10, 11). With the increasing incidence of GDM yearly, the number of GDM cases is enormous. Reducing the harm to the pregnancy outcome of GDM through managing pregnancy is challenging. However, studies identifying pregnant women with high-risk GDM are still limited.

Multiple inflammatory factors and immune regulatory factors are disordered in GDM patients (12). Recent studies have highlighted the potential role of inflammatory indicators in the pathogenesis of GDM and its complications (13–15). Interestingly, some studies have found that the blood neutrophil-lymphocyte ratio (NLR) is a valuable diagnostic and prognostic biomarker for clinical inflammatory diseases, including preterm labor and GDM (16, 17). Fortunately, the systemic immune-inflammation index (SII) and systemic inflammation response index (SIRI) are novel and stable inflammatory predictors that can respond to the local immune status and systemic inflammation with peripheral blood (18, 19). Moreover, recent studies have demonstrated that SII and SIRI can better reflect the chronic inflammatory state than NLR and other inflammatory indicators (20, 21). Therefore, they can serve as valuable biomarkers of systemic inflammation with more effectiveness and stability. However, the relationships of SII and SIRI with the maternal-neonatal outcome in patients with GDM remain unknown.

This study analyses the relationships between the inflammatory markers and the pregnancy outcomes in patients with GDM. We aimed to evaluate whether the inflammatory markers with the mother’s peripheral blood can be valuable predictors for pregnancy outcomes in GDM with non-invasive.

## Materials and methods

### Participants

This is a retrospective study. It analyzed 3,229 pregnancies with GDM from Fujian Provincial Maternity and Child Health Hospital, Affiliated Hospital of Fujian Medical University (FMCH) from January 2014 to January 2020. All women must meet the following criteria: (1) GDM was confirmed at 24 weeks of Oral Glucose Tolerance Test (OGTT); and (2) Regular prenatal examination in our hospital; Pregnant women were excluded due to the following criteria: (1) multi-fetal pregnancies, (2) severe medical and surgical complications; (3) fetal anomalies; and (4) incomplete clinical data. Detailed data with pregnancy outcomes were collected from computerized obstetric records and neonatal databases. The Hospital Ethics Committee of Fujian Provincial Maternity and Children’s Hospital, an affiliated hospital of Fujian Medical University, approved the study (2021KLRD634).

### Definition

The diagnostic criteria for GDM should meet the following 1 or more than 2 terms according to the results of 75 g OGTT test at 24 to 28 weeks: (1) FPG: 5.1 to 6.9 mmol/L; (2) 1 h glucose: 10.0 mmol/L; and (3) 2 h glucose: 8.5 to 11.0 mmol / L. Good pregnancy outcome was defined as the neonate and mother being discharged from the hospital without identifiable complications. Poor pregnancy outcomes resulted in miscarriage, intrauterine death, and neonatal and maternal complications. Significant neonatal complications were macrosomia, low birth weight infant (LBW), neonatal asphyxia and neonatal intensive care unit (NICU) admission. The maternal complications included gestational hypertension, abnormal amniotic fluid volume, preterm premature rupture of membranes (PPROM), postpartum hemorrhage, and placental abruption.

### Blood cell count assay

The peripheral blood samples were collected during the first trimester. Cell counts, such as White blood cells (WBC), neutrophils, lymphocytes, monocytes, and platelets, were taken with flow cytometry (XE-3000, SYSMES, Kobe, Japan). The SII (platelet count × neutrophil count/lymphocyte count) and SIRI (monocyte count × neutrophil count/lymphocyte count) were calculated with absolute counts (22, 23).

### Statistical analysis

Counting data were expressed as numbers (%). Measurement data for normal and skew distribution are expressed as mean, standard deviation, and median, respectively. All analyses were calculated by SPSS version 26.0 (IBM, Armonk, NY, United States). The Chi-2 test or Fisher’s exact test was used to compare the relationship between pregnancy outcome and clinical characteristics. The area under the receiver operating characteristic (ROC) curve (AUC) was used to calculate the predictive efficiency of inflammatory markers (SII and SIRI) with pregnancy outcomes (24). Also, the Spearman rank correlation coefficient was used to assess relationships between inflammatory markers (SII and SIRI) and pregnancy outcomes. Multiples logistic regression analysis was performed to predict the poor outcomes in patients with GDM. In all statistical tests, the differences were considered statistically significant at *p*-values<0.05.

## Results

### The clinical characteristic of maternal-neonatal outcomes

Finally, 3,229 women with GDM were included. The maternal and neonatal clinical data are shown in Table 1. The mean age of the pregnancy was 31.75 ± 4.72 years, and the mean base body mass index (BMI) was21.80 ± 3.02 (kg/m^2^). And then, the mean gestational age at delivery was 38.99 ± 1.66 weeks. Of the pregnancies, some pregnancy suffered complications, such as postpartum hemorrhage, cesarean delivery, gestational hypertension, placental abruption. As for neonatal outcomes, there are 12 (0.40%) newborns died. The mean birth weight was 3279.83 ± 493.85 g. Most baby good prognosis.

**Table 1:** Clinical characteristics of the study population.

Characteristics		Number of cases (%)
Mother	Maternal age (years)	31.75 ± 4.72
	BMI (kg/m^2^)	21.80 ± 3.02
	Lean	165 (5.10)
	Normal	1,668 (51.70)
	Overweight	1,152 (31.70)
	Obesity	244 (7.60)
	Gravida	2 (1–8)
	Parity	1 (0–3)
	PPROM	895 (27.70)
	GA at delivery (weeks)	38.99 ± 1.66
	Cesarean delivery	1,344 (41.60)
	Gestational hypertension	181 (5.60)
	Placental abruption	48 (1.50)
	Postpartum hemorrhage	56 (1.70)
	Abnormal amniotic fluid volume	106 (3.30)
	WBC (×10^9^/L)	9.90 ± 2.89
	Neutrophils (×10^9^/L)	7.47 ± 2.69
	Lymphocyte (×10^9^/L)	1.61 ± 0.54
	Monocyte (×10^9^/L)	0.66 ± 0.24
	Platelet (×10^9^/L)	208.62 ± 54.65
	SII (×10^9^/L)	1022.76 ± 574.52
	SIRI (×10^9^/L)	3.77 ± 2.63
Newborn	GA at delivery (week)	
	<28 wk	13 (0.40)
	≥28 to <37 wk	210 (6.50)
	37 ≥ wk	3,006 (93.10)
	Birth weight (g)	3279.83 ± 493.85
	LBW	167 (5.20)
	Macrosomia	188 (5.80)
	Mortality	12 (0.40)
	NICU admission	270 (8.40)
	Neonatal asphyxia	20 (0.60)

Continuous variables are presented as mean ± SD (range) and categorical variables as *n* (%). Recurrent abortion was defined as at least three spontaneous abortions. BMI, body mass index; GA, gestational age; PPROM, preterm premature rupture of membranes; WBC, white blood cell; SII, systemic immune inflammation index; SIRI, systemic inflammation response index; LBW, low birth weight infant; SGA, small for gestational age; NICU, neonatal intensive care unit.

### The inflammatory markers are predictive risk factors for pregnancy outcomes

**Figure 1:**
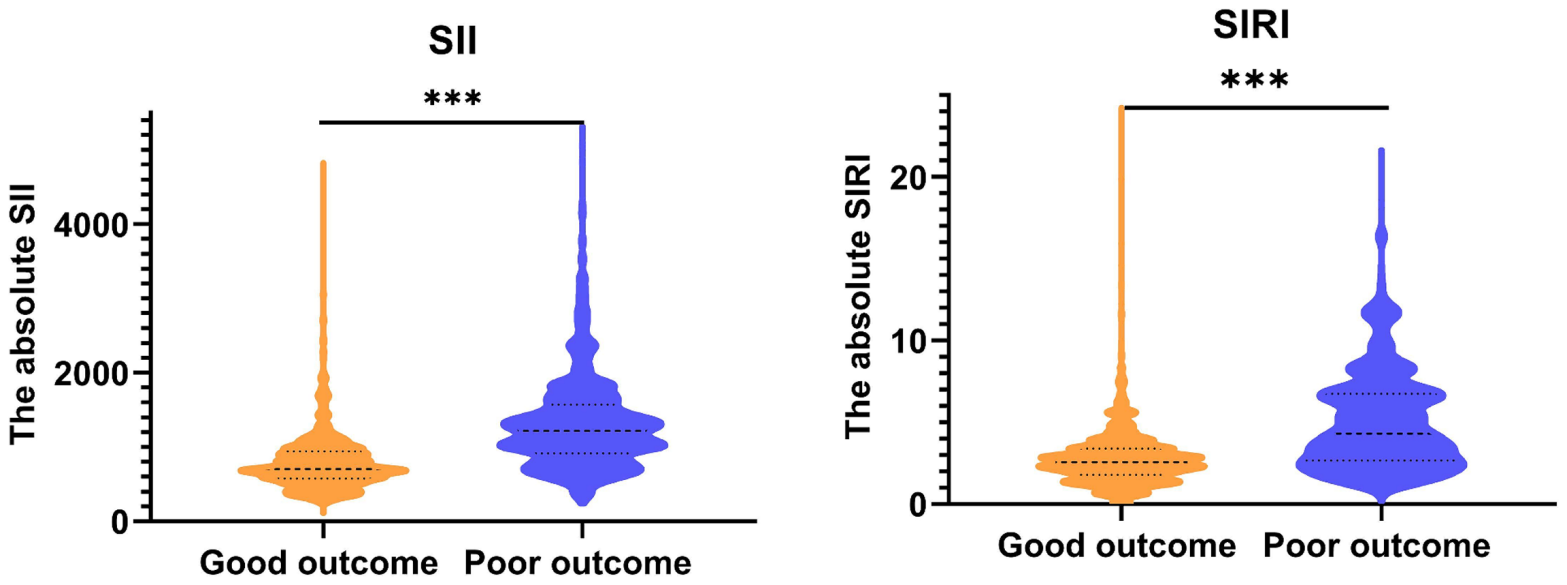
The expression of SII and SIRI among all participants. SII, systemic immune inflammation index; SIRI, systemic inflammation response index.

Finally, 1852 (57.36%) participants suffered good pregnancy outcomes. This study revealed that the maternal age, the baseline BMI (kg/m^2^), and the times of parity were significantly different between the good pregnancy outcomes and poor groups (all *p* < 0.05). The lymphocyte, SII, and SIRI levels significantly increased in poor pregnancy outcomes groups (Table 2). Also, the results shown that the average SII in good groups and poor groups were 802.57 ± 404.08 × 109/L and 1318.92 ± 633.76 × 109/L. Similarly, the average SIRI in good groups and poor groups were 2.79 ± 1.75 × 109/L and 5.09 ± 3.03 × 109/L (Figure 1).

**Table 2:** The association between inflammatory markers and pregnancy outcomes in patient with GDM.

Variable	Good outcome (*N* = 1852) *n* (%)	Poor outcome (*N* = 1,377) *n* (%)	*p*‑value
Maternal age (years)	31.47 ± 4.74	31.95 ± 4.69	0.005
BMI (kg/m^2^)	22.02 ± 3.08	21.63 ± 2097	<0.001
Gravida Median (min-max)	2 (1–8)	2 (1–8)	0.490
Parity Median (min-max)	0 (0–3)	1 (0–3)	0.030
WBC (×10^9^/L)	9.82 ± 2.76	10.01 ± 3.05	0.062
Neutrophils (×10^9^/L)	7.49 ± 2.58	7.44 ± 2.83	0.592
Lymphocyte (×10^9^/L)	1.59 ± 0.54	1.65 ± 0.54	0.005
Monocyte (×10^9^/L)	0.67 ± 0.24	0.66 ± 0.23	0.214
Platelet (×10^9^/L)	208.53 ± 53.27	208.72 ± 56.48	0.923
SII (×10^9^/L)	802.57 ± 404.08	1318.92 ± 633.76	<0.001
SIRI (×10^9^/L)	2.79 ± 1.75	5.09 ± 3.03	<0.001

Categorical data were analyzed using chi-squared tests, and continuous data with *t*-tests. BMI, body mass index; WBC, white blood cell count; SII, systemic immune inflammation index; SIRI, systemic inflammation response index.

### The relationships between the inflammatory markers and maternal-neonatal outcomes

Table 3 revealed that inflammatory markers based on maternal blood, such as WBC, lymphocyte, neutrophils, monocyte, platelet counts, SII, and SIRI, were significantly related to pregnancy outcomes. It suggested higher WBC counts related to a higher cesarean delivery rate, postpartum hemorrhage and gestational hypertension. The higher neutrophil counts are related to a higher rate of cesarean delivery and postpartum hemorrhage. The higher lymphocyte counts were related to a higher rate of the bulging membrane, gestational hypertension and a lower rate of cesarean delivery. Moreover, higher SII and higher SIRI levels are related to a higher rate of PPROM, a cesarean delivery, gestational hypertension, placental abruption and abnormal amniotic fluid volume. Similarly, it revealed that mothers with higher WBC counts, higher neutrophils counts, lower lymphocyte counts, higher SII and higher SIRI levels related to lower birth weight, a higher rate of LBW, a higher rate of macrosomia, and a higher mortality rate, a higher rate of NICU admission, and a higher rate of neonatal asphyxia.

**Table 3:** Relationships between the inflammatory markers and maternal-neonatal outcomes.

Variable		WBC	Neutrophils	Lymphocyte	Monocyte	Platelet	SII	SIRI
PPROM	*r*-value	0.120	−0.017	0.061	−0.027	0.009	0.229	0.246
	*p*-value	0.501	0.331	<0.001	0.129	0.615	<0.001	<0.001
Cesarean delivery	*r*-value	0.099	0.126	−0.158	0.069	−0.006	0.165	0.162
	*p*-value	<0.001	<0.001	<0.001	<0.001	0.747	<0.001	<0.001
Postpartum hemorrhage	*r*-value	0.112	0.096	0.006	0.025	−0.049	−0.007	0.014
	*p*-value	<0.001	<0.001	0.748	0.158	0.006	0.699	0.410
Gestational hypertension	*r*-value	0.058	0.010	0.040	0.025	0.017	0.079	0.113
	*p*-value	0.001	0.582	0.025	0.158	0.329	<0.001	<0.001
Placental abruption	*r*-value	0.034	0.019	−0.004	0.024	0.013	0.047	0.036
	*p*-value	0.051	0.270	0.828	0.172	0.462	0.007	0.043
Abnormal amniotic fluid volume	*r*-value	0.002	−0.011	−0.019	0.002	−0.009	0.062	0.068
	*p*-value	0.898	0.548	0.290	0.896	0.613	<0.001	<0.001
Birth weight	*r*-value	−0.099	−0.072	−0.087	−0.006	−0.062	0.068	0.095
	*p*-value	<0.001	<0.001	<0.001	0.737	<0.001	<0.001	<0.001
LBW	*r*-value	0.083	0.072	0.058	0.024	0.025	0.156	0.146
	*p*-value	<0.001	<0.001	0.001	0.169	0.157	<0.001	<0.001
Macrosomia	*r*-value	−0.018	−0.008	−0.044	0.002	−0.001	0.095	0.112
	*p*-value	0.311	0.637	0.012	0.894	0.951	<0.001	<0.001
Mortality	*r*-value	−0.004	0.004	−0.017	0.008	0.028	0.022	0.036
	*p*-value	0.819	0.832	0.325	0.652	0.107	0.206	0.041
NICU admission	*r*-value	0.068	0.040	0.004	0.026	0.002	0.054	0.055
	*p*-value	<0.001	0.024	0.819	0.145	0.889	0.002	0.002
Neonatal asphyxia	*r*-value	0.048	0.027	−0.035	0.025	−0.019	0.045	0.086
	*p*-value	0.006	0.122	0.050	0.149	0.282	0.010	<0.001

Analysis was performed using Spearman’s rank correlation analysis. PPROM, preterm premature rupture of membranes; WBC, white blood cell; SII, systemic immune inflammation index; LBW, low birth weight infant; SIRI, systemic inflammation response index NICU, neonatal intensive care unit.

### Predictive value of inflammatory markers for pregnancy outcomes with GDM

**Figure 2:**
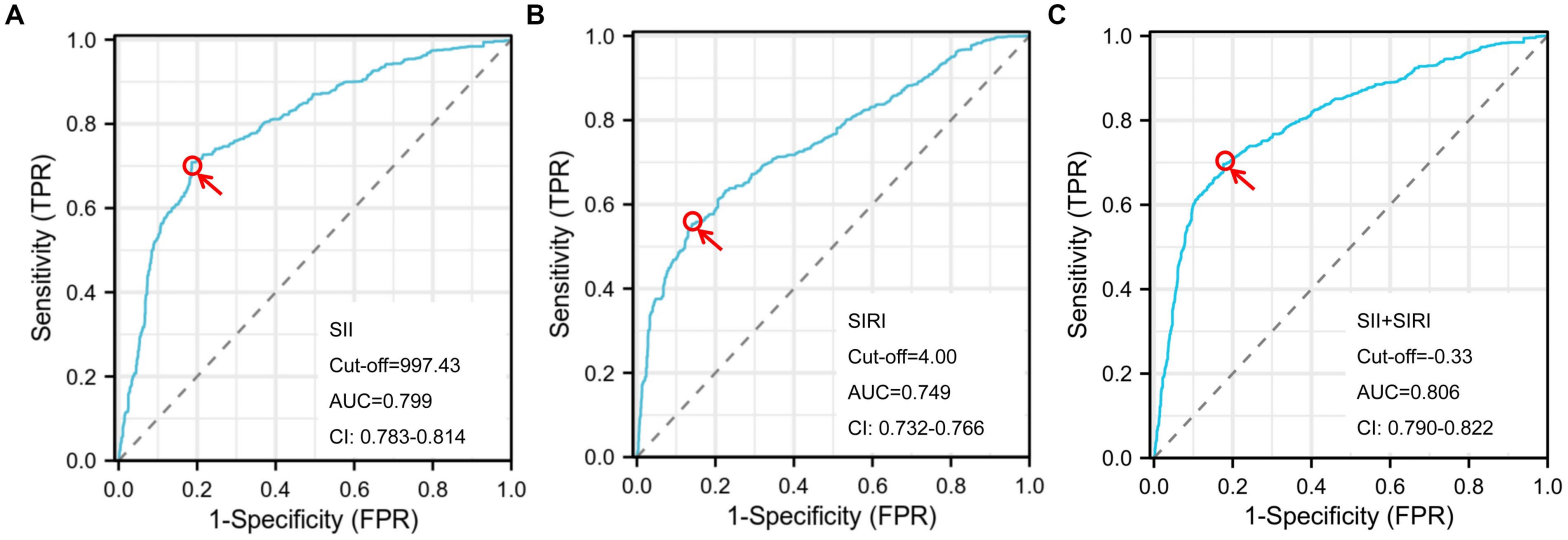
Cut-off, the optimal SII level, SIRI level and combined model is used to predict pregnancy outcome with GDM according to the ROC curve. SII, systemic immune inflammation index; SIRI, systemic inflammation response index; SII+SIRI, the SII, SIRI and combined model; AUC, the area under the ROC curve; ROC, receiver operator characteristic; CI, confidence interval.

ROC analyses of SII and SIRI were used to predict the pregnancy outcomes with GDM (Figures 2A,B). We found that the AUC for the SII level (0.799) was better than the SIRI level (0.749). Moreover, the optimal cut-off of the SII and SIRI levels were 997.43 and 4.00. Furthermore, we revealed that the combination of SII and SIRI had the highest AUC (0.806) (Figure 2C). The sensitivity and specificity of SII, SIRI and combined model were 70.88/55.34/69.60% and 81.37/82.91 /84.34%. In addition, the PPV and NPV of the SII and SIRI were 73.88/74.49/74.53% and 78.98/72.12/79.48% (Table 4).

**Table 4:** Diagnostic accuracy of the inflammatory markers for the pregnancy outcomes with GDM.

	Sensitivity	Specificity	PPV	NPV
SII	70.88 (68.48–73.28)	81.37 (79.60–83.14)	73.88 (71.51–76.25)	78.98 (77.16–80.81)
SIRI	55.34 (52.71–57.96)	82.91 (84.32–86.49)	74.49 (71.82–77.16)	72.12 (70.25–73.99)
Combined model	69.60 (67.17–72.03)	84.34 (80.61–88.08)	74.53 (72.15–76.92)	79.48 (76.66–81.31)

PPV, positive predictive value; NPV, negative predictive value; SII, systemic immune inflammation index; SIRI, systemic inflammation response index.

As shown in Table 5, the independent risk factors associated with pregnancy outcomes with GDM were used by multivariable logistic regression analysis. The results presented that the SII had the highest OR [OR = 6.957; 95% CI (5.715–8.468)], followed by SIRI [OR = 2.948; 95% CI (2.382–3.649)], the WBC counts [OR = 1.930; 95% CI (0.901–2.960)], the lymphocyte counts [OR = 1.668; 95% CI (1.412–1.970)], and baseline BMI [OR = 1.050; 95% (1.021–1.080)].

**Table 5:** Independent risk factors for predicting the poor outcomes in patient with GDM.

Factors	OR	95% CI	*p*-value
Maternal age (years)	0.985	0.966–1.006	0.159
Parity	0.713	0.600–0.848	0.006
BMI (kg/m^2^)	1.050	1.021–1.080	0.001
WBC (×10^9^/L)	1.930	0.901–2.960	<0.001
Lymphocyte (×10^9^/L)	1.668	1.412–1.970	<0.001
SII (×10^9^/L)( ≥ 997.430)	6.957	5.715–8.468	<0.001
SIRI (×10^9^/L)( ≥ 9.998)	2.948	2.382–3.649	<0.001

BMI, body mass index; WBC, white blood cell count; SII, systemic immune inflammation index; SIRI, systemic inflammation response index; CI, confidence interval.

## Discussion

In recent years, people have improved their economic level, changed their lifestyle and diet, increased the overweight and obese population, and policy changes. In general, the prevalence of GDM in China shows an apparent upward trend, which brings heavy pressure on diagnosis and treatment and economic burden to society. Moreover, GDM will affect the health of two generations and even the population’s quality (3, 4). Therefore, the emphasis on early detection of high-risk individuals and early prevention and intervention measures will have profound significance for reducing adverse pregnancy outcomes in pregnant women with GDM and is currently a priority in obstetrics. However, there are currently no clinically effective measures for the early identification of pregnant women with high-risk GDM. Therefore, there is an urgent need to study reliable indicators to predict the pregnancy outcome with GDM and provide guidance measurements to improve the outcome.

In this study, 1852 (57.36%) GDM participants suffered good pregnancy outcomes. It suggests that nearly half of GDM women had a poor pregnancy outcome. And then this study reported that the maternal age, the baseline BMI (kg/m^2^) and the times of parity were significantly different between the good pregnancy outcomes and poor groups, which is similar to other studies (25, 26). Our results indicate that the cesarean delivery proportion of this study population was 41.60%, which are consistent with other results (27). Such a high proportion may be due to the special population of GDM. Also, this study demonstrated that those inflammatory markers, such as the lymphocyte counts and the baseline SII and SIRI based on peripheral blood, were significantly related to the pregnancy outcome. Moreover, it revealed that the AUC for the SII level (0.799) was better than the SIRI level (0.749). Moreover, the optimal cut-off of the SII and SIRI levels were 997.430 and 3.998. Finally, it demonstrated the combination of SII and SIRI had the highest AUC (0.806). Multivariable logistic regression analysis found that the level of SII and SIRI were independent risk factors for poor pregnancy outcomes in women with GDM. These non-invasive markers could assist in making clinical decisions to identify high-risk pregnant women with GDM early.

The pathogenesis of GDM remains not fully understood, but insulin resistance and the relative insufficiency of insulin secretion are essential features of GDM (16). Inflammation plays an important role in the pathogenesis of GDM (28). In recent years, researchers have been more and more deeply exploring the relationship between conventional blood inflammatory markers and diseases and found that many of these factors and their derived indicators can be used as emerging indicators to predict the occurrence and development of diseases and evaluate the prognosis. Some case–control studies found that CRP, IL-6 and TNF-α in pregnant women with GDM were significantly increased in early pregnancy, which indicated that inflammatory indicators are closely related to GDM (13, 14). Studies have reported that neutrophil activity was increased in GDM patients, and neutrophil extracellular traps (NETs), a reticulate ultrastructure released to the extracellular cell after neutrophil activation, was significantly increased compared with normal pregnant women (29). A recent study revealed that NETs also could inhibit the proliferation, migration, invasion, and angiogenesis of placental extravillous trophoblast cells (30). Also, it can alter the biological functions of placental cells and cause adverse perinatal outcomes (31). Monocytes account for about 20% of the total leukocytes at the site of placental implantation and are the primary mediators of placental tissue remodeling (32). Recent studies have found a clear association between monocytes and the onset of gestational diabetes, pointing to a significantly increased risk of gestational diabetes mellitus when the monocyte count is 0.4 × 10^9^/L (33). Importantly, some studies have demonstrated that the blood NLR is an important biomarker for clinical inflammatory diseases, such as preterm labor and GDM (16, 17). However, unfortunately, these indicators can only reflect the body’s situation from different aspects.

Some studies have reported that SII and SIRI are comprehensive biomarkers based on peripheral blood that more fully reflect the state of local immunity and systemic inflammatory response (18, 19). SII is a new indicator of systemic immune inflammation, which is associated with many tumors and inflammatory diseases, including pancreatitis and novel coronavirus pneumonia (34–36). Elevated SII levels suggest a poor prognosis (37). Similarly, SIRI is a novel inflammatory index. And many reaches have reported that it can be a valuable prognostic predictor in patients with tumors. As a predictor, it has certain advantages over a traditional inflammatory index, tumor markers, and pathological tumor stages (38–40). It demonstrated that a higher SIRI level is associated with poor prognosis. Also, the biomarkers SII and SIRI are less cost-effective and non-invasive, so they are gradually applied to the clinics (41). Moreover, some studies have revealed that SII and SIRI can better reflect the chronic inflammatory reaction than NLR and other inflammatory indicators (21).

This study also has some limitations. Firstly, this is a retrospective study. Therefore, a prospective study is necessary in the future. Secondly, the study focused on clinics and did not explore the mechanism by which inflammatory indicators influence pregnancy outcomes. Therefore, further studies are needed to explore the underlying molecular mechanisms.

In conclusion, this study revealed that the baseline SII and SIRI levels with mothers emerged as important biochemical markers for predicting the pregnancy outcome of women with GDM. Moreover, our data proved that the SII level ≥ 997.430 and the SIRI level ≥ 3.998 indicated poor pregnancy outcomes. We demonstrated the combined measurement of baseline SII and SIRI levels as a suitable and practical biomarker to predict pregnancy outcomes in women with GDM by non-invasive methods. These results may offer additional clinical information, which helps identify high-risk pregnant women with GDM early.

## Data availability statement

The raw data supporting the conclusions of this article will be made available by the authors, without undue reservation.

## Ethics statement

The studies involving humans were approved by the Hospital Ethics Committee of Fujian Provincial Maternity and Children’s Hospital, an affiliated hospital of Fujian Medical University, approved the study (2022KYLLR03050). The studies were conducted in accordance with the local legislation and institutional requirements. The ethics committee/institutional review board waived the requirement of written informed consent for participation from the participants or the participants’ legal guardians/next of kin because This study is a retrospective study, the data are anonymous, and the requirement for informed consent was therefore waived.

## Author contributions

XX: Funding acquisition, Writing – original draft. YL: Writing – original draft. ZC: Data curation, Formal analysis, Investigation, Writing – original draft. LL: Data curation, Formal analysis, Investigation, Supervision, Writing – original draft. YZ: Data curation, Formal analysis, Methodology, Writing – original draft. JY: Conceptualization, Project administration, Visualization, Writing – review & editing.
